# 

**Published:** 1936

**Authors:** 


					Ai
Vol. Lin. No. 200. / M?c
v>i%'
' P '. m - , :
1936.
60s1* v
w
JA
ii i
The B
vA;v-, :
PUBLISHED TJNDEB THE AUSPICES OF
THE BRISTOL MEDICO-CHIRURGICAL SOCIETY.
Editor: J. A. NIXON, C.M.G., M.D., F.R.C.P.
WITH WHOM ABB ASSOCIATED
' ? ^ -V;""' " ' ? '>1 ? ? v* ' ' '? ? ?!
E. W. HEY GROVES, D.So., M.S., P.R.0.8.
A. E. ILES, O.B.E., M.B., B.S., P.R.0.8.
A. RENDLE SHORT, M.D., B.S., B.Sc., FJl.0.8.
E. WATSON-WILLIAMS, M.O., Oh.M., P.R.0.8., Atsitlani Editor.
Editorial Secretary: A. L. PLEMMINQ, M.B., Ob.B.
BRISTOL: J. W. ARROWSMXTH LTD., 11 QUAY STREET
London: J. W. Abbowsmith (London) to.
Fox wood House, Pox wood Place, High Holbobn, W.C. 1
Price Three Shillings net.
^wmbbmi?i

				

